# Something New about Oxygen

**Published:** 1877-11

**Authors:** 


SOMETHING NEW ABOUT OXYGEN.—Recent investigations have disclosed the singular fact, that oxygen under high pressure, rapidly destroys all living beings and organic compounds. All the varied phenomena of fermentation, in which the chemical action depends upon the presence of living organisms, are completely arrested by the action of compressed oxygen, even if exerted for only a brief time; while fermentations due to dissolved matter, like diastase, perfectly resist its influence. M. Bert, to whom this curious discovery is due, has found a practical application of it in the field of physiological research. The ripening of fruits is arrested by exposure to compressed oxygen, and hence it must arise from cellular evolution. The poison of the scorpion, on the other hand, whether liquid, or re-dissolved in water, entirely resists the action of the compressed gas. Such poisons evidently owe their power to chemical compounds akin to the vegetable alkaloids. Fresh vaccine matter, subjected for more than a week to oxygen under a pressure equal to fifty atmospheres, retained its virtue; from which it would appear that the active principle in vaccine matter is not certain living organisms or cells, as some have supposed. The virus of glanders, after similar treatment, quickly infected horses inoculated with it; and carbuncular blood, though freed from bacteria, was found to retain its dangerous properties after the same test. These must, therefore, be put in the same class with vaccine matter.

If these results are confirmed by further investigations, the discovery is certainly a most important one, and will lead to the settlement of many disputed questions in physiological chemistry.—*Journal of Chemistry.*

